# Effect of Fitzpatrick Skin Type Prompting on Diagnostic Accuracy in Multimodal Large Language Models: A Within-Image Experimental Study

**DOI:** 10.3390/bioengineering13080932

**Published:** 2026-08-18

**Authors:** Manoj Bhagwat, Tyler Wittles, Jeffery Tan, Joshua Mijares, Neil K. Jairath, Syril Keena T. Que

**Affiliations:** 1 Department of Dermatology, Indiana University School of Medicine, Indianapolis, IN 46202, USA; 2 Ronald O. Perelman Department of Dermatology, New York University Grossman School of Medicine, New York, NY 10016, USA

**Keywords:** diagnostic accuracy, ChatGPT, GPT-5.2, Gemini, large language models, artificial intelligence, prompt engineering, Fitzpatrick skin type, Diverse Dermatology Images, skin of color

## Abstract

Multimodal large language models are increasingly used for dermatologic queries, but whether Fitzpatrick skin type (FST) labels affect diagnostic accuracy is unknown. We evaluated 656 biopsy-confirmed photographs from the Diverse Dermatology Images (DDI) dataset under no-FST, DDI-concordant FST, and two DDI-discordant FST conditions using ChatGPT 5.2 Edu and Gemini 3.1 Pro browser configurations (5248 evaluations). Confirmed malignant diagnosis omission from the top three differential diagnoses (“lethal miss”) was a prespecified exploratory outcome. Gemini had higher ordinal accuracy than ChatGPT (odds ratio 2.32; 95% confidence interval 2.01–2.67; false discovery rate-adjusted *p* < 0.001). No FST prompt condition significantly improved accuracy over no-FST prompting; an equal-image-weighted sensitivity analysis yielded similar results. Among malignant images eligible for extreme discordance, extreme DDI-discordant prompting was associated with more ChatGPT lethal misses than no-FST prompting (paired odds ratio 5.0; *p* = 0.043); Gemini showed no significant paired shift (*p* = 1.00). Post hoc binary malignancy detection showed low no-FST specificity for ChatGPT (42.5%) and Gemini (22.9%), indicating frequent overcalling. Gemini showed lower no-FST accuracy for malignant FST V–VI images than for FST I–II and III–IV images. FST prompting provided no measurable diagnostic benefit. Neither configuration supports autonomous dermatologic use.

## 1. Introduction

Artificial intelligence (AI) applied to dermatologic image analysis has advanced considerably over the past decade, with some systems demonstrating strong diagnostic performance in constrained benchmark settings [1]. Beyond conventional classifiers and segmentation systems, recent work has evaluated dermatology-focused and general-purpose multimodal large language models (LLMs) for diagnosis and management [2,3,4,5]. A consistent finding across this literature, however, is that some dermatology AI systems underperform on darker skin tones, and recent multimodal dermatology AI work has also reported management-plan quality degradation across Fitzpatrick skin type (FST) groups [2,6]. These disparities have been partly attributed to underrepresentation of darker skin tones in training and evaluation data; related skin tone effects have been documented in both image classification and segmentation architectures [6,7].

Across other clinical imaging domains, general-purpose multimodal LLMs have demonstrated heterogeneous and frequently limited visual diagnostic performance. In a 515-image multianatomic radiology evaluation, GPT-4V reliably identified imaging modality and anatomic region but showed limited diagnostic classification performance, with pooled sensitivity of 78% and specificity of 32.3% in binary tasks and high false-positive rates [8]. On the American College of Radiology in-training examination questions, GPT-4V accuracy was substantially higher for text-only than image-based questions (81.5% vs. 47.8%) [9]. In 401 neuroradiology cases, multimodal LLM top-3 accuracy ranged from 29.9% to 49.4%, compared with 80.3% and 68.3% for two neuroradiologists, and performance declined further when textual clinical history was unavailable [10]. A systematic review of 18 studies found that multimodal vision-language models generally outperformed unimodal inputs, but standalone comparisons with physicians were conflicting; most included studies had high risk of bias, and no firm recommendation could be made for standalone clinical use [11]. Taken together, these results indicate that strong performance on text-rich or curated benchmarks should not be read as evidence of reliable autonomous image interpretation.

Recent studies in Bioengineering and related MDPI journals report similar limitations across clinical imaging tasks. In periapical radiographs, ChatGPT-5 achieved 87.5% sensitivity but 12.5% specificity and 50.0% accuracy, over-identifying pathology [12]. In a seven-model radiology study, accuracy ranged from 8.1% to 29.2%, while hallucinations occurred in 74.4% of assessments, and clinical context increased accuracy but also text dependence [13]. In a five-run hand-fracture study, mean accuracy across models ranged from 33.8% to 64.3%; one model showed high consistency despite low accuracy, while another was markedly unstable [14]. In brain-metastasis MRI, GPT-4o and Claude Sonnet 3.5 achieved 100% sensitivity but only 8% and 4% specificity and over-reported lesions in 12% of cases [15]. Although limited to a single deliberately selected head CT, a five-model evaluation documented one fundamental diagnostic error and clinically meaningful intermodel disagreement, and its authors cautioned against autonomous interpretation [16]. Though tasks and modalities differ, low specificity, hallucinated findings, and instability recur across these evaluations as barriers to autonomous image interpretation.

Multimodal LLMs differ from earlier text-based or vision-only systems in that they integrate visual and textual inputs simultaneously. Because these models process both the clinical image and the text of the prompt simultaneously, auxiliary contextual information provided in the prompt can influence diagnostic outputs. Prior text-based LLM studies have shown that demographic descriptors in prompts shift clinical recommendations [17,18], and recent evaluations have documented implicit-bias patterns affecting downstream medical decision tasks [19]. This creates a distinct risk that has not been systematically evaluated: demographic descriptors included in prompts, such as skin tone labels, may shape model outputs whether or not that information is accurate. Given that skin tone assessment is not standardized and skin tone metadata are often absent, inferred, or inconsistently documented, the introduction of inaccurate contextual metadata is foreseeable [20,21,22,23]. No prior within-image dermatology study appears to have deliberately varied whether an FST label was absent, concordant with the dataset-assigned group, adjacent-discordant, or extreme-discordant.

FST is the most used skin tone or skin-phototype label in dermatologic AI image work [6,22], making it the natural reference frame for skin tone prompting in multimodal models. Despite this widespread use, FST has well-documented limitations as a skin tone descriptor [20,22]. It is frequently repurposed as a proxy for skin color or race/ethnicity and can be inferred or assigned inconsistently in dataset labeling and clinical documentation [20,21,22,23]. Furthermore, FST aligns poorly with objective skin color measurement [21,22] because it was originally designed around ultraviolet reactivity and photosensitivity rather than pigmentation [24,25]. These limitations mean that FST labels attached to clinical images are often unreliable proxies for the underlying skin tone characteristics relevant to AI diagnostic performance.

The growing public availability of multimodal LLMs has led to substantial use for health-related information seeking. Every week, ChatGPT alone has more than 230 million people globally who ask health- and wellness-related questions [26]. Understanding how prompt-level metadata affects diagnostic outputs in these systems is therefore a practical safety question. Inputting an FST in a prompt to improve AI-assisted diagnosis may, if that label is incorrect, inadvertently degrade the quality of the model’s output.

We conducted a controlled, within-image paired experiment using the Diverse Dermatology Images (DDI) dataset to test whether FST metadata improves diagnostic accuracy in multimodal LLMs and whether DDI-discordant FST labels can increase errors, including complete omission of confirmed malignancies from the top three differential diagnoses. This within-image design controlled for lesion type, image quality, and diagnosis. Two flagship multimodal LLMs available at the time of data collection, ChatGPT 5.2 and Gemini 3.1 Pro, were evaluated using the same source images, visible prompt text, and scoring procedures across all 656 biopsy-confirmed photographs in the dataset [6]. This design isolates the effect of prompt-supplied FST metadata under controlled conditions but does not constitute clinical validation of either configuration.

## 2. Materials and Methods

### 2.1. Study Design and Dataset

This is a non-human subjects research study using a controlled, within-image paired experimental design. De-identified photographs and associated metadata were obtained from the DDI dataset [6]. The DDI is a publicly available, biopsy-confirmed collection curated by the Stanford University School of Medicine [6]. The dataset comprises 656 images from 570 patients sourced across multiple Stanford clinics, providing a degree of clinical heterogeneity while remaining limited by its single-center origin.

Available metadata were limited to three labels per image: a histopathologically confirmed diagnosis, binary malignancy status (malignant vs. benign), and a dermatologist-assigned FST group (I–II, III–IV, or V–VI). FST labels were determined through chart review of in-person visits and consensus by two board-certified dermatologists [6]. Histopathologic diagnosis served as the reference standard for diagnostic scoring, whereas FST was treated as DDI-assigned metadata rather than independently verified ground truth. We did not independently reassess FST using colorimetry, another objective skin color scale, or additional dermatologist review, and per-image assignment uncertainty was unavailable. The DDI data available for this study did not include race, ethnicity, sex, gender, or anatomic location metadata, which constrained bias analyses to FST-related comparisons. The released metadata grouped FST as I–II, III–IV, and V–VI; differences within each paired category could therefore not be examined. The image distribution was 208 images (159 benign, 49 malignant) for FST I–II, 241 (167 benign, 74 malignant) for FST III–IV, and 207 (159 benign, 48 malignant) for FST V–VI [6].

Images were submitted as provided in the DDI dataset without preprocessing, augmentation, normalization, resizing, or enhancement. No synthetic images were generated or used. Because no model was trained, fine-tuned, or internally modified, no training, validation, or test partitions were created. Evaluation was limited to the DDI dataset, and no rebalancing, resampling, or class weighting was performed. This protocol was reviewed and approved by the Indiana University School of Medicine Institutional Review Board (approval #30423).

### 2.2. Models Evaluated

We evaluated OpenAI’s ChatGPT 5.2 and Google’s Gemini 3.1 Pro, prominent browser-accessible multimodal models available at the time of data collection [27,28]. ChatGPT 5.2 was accessed via an institutional Edu license in the standard browser interface, not using the Instant, Thinking, or Pro variants. Gemini 3.1 Pro was queried through its standard browser interface. The same source image files and visible prompt text were submitted to both systems. Each system was accessed through its own browser interface; accordingly, the comparison applies to the deployed model–interface configurations rather than to isolated base models. Post-upload image resolution, platform compression, hidden system instructions, safety settings, sampling parameters, and backend routing were not observable or controllable through either interface. Model names and collection dates were recorded in the study protocol. Temporary Chat mode was used, and refreshing the page opened a new temporary conversation without the prior visible chat context. Data were collected from ChatGPT between 5 February and 1 March 2026 and from Gemini between 19 February and 15 March 2026.

ChatGPT is widely used for health-related queries, and both ChatGPT and Gemini are browser-accessible multimodal LLMs increasingly evaluated in dermatology [3,4,5,26]. The prior dermatology LLM literature has focused heavily on ChatGPT, with Gemini-family models appearing in more recent comparative evaluations [3,4,5]. These two models were selected because browser-based access reflects a common non-API mode of use. Although version-specific performance may change, the within-image protocol is model-agnostic and can be repeated with other multimodal systems that accept image and text inputs. Both models were treated as black-box systems without access to or control over internal parameters such as temperature. Outputs may therefore exhibit stochastic variability, and exact response replication cannot be guaranteed.

### 2.3. Prompt Conditions

**Figure 1:**
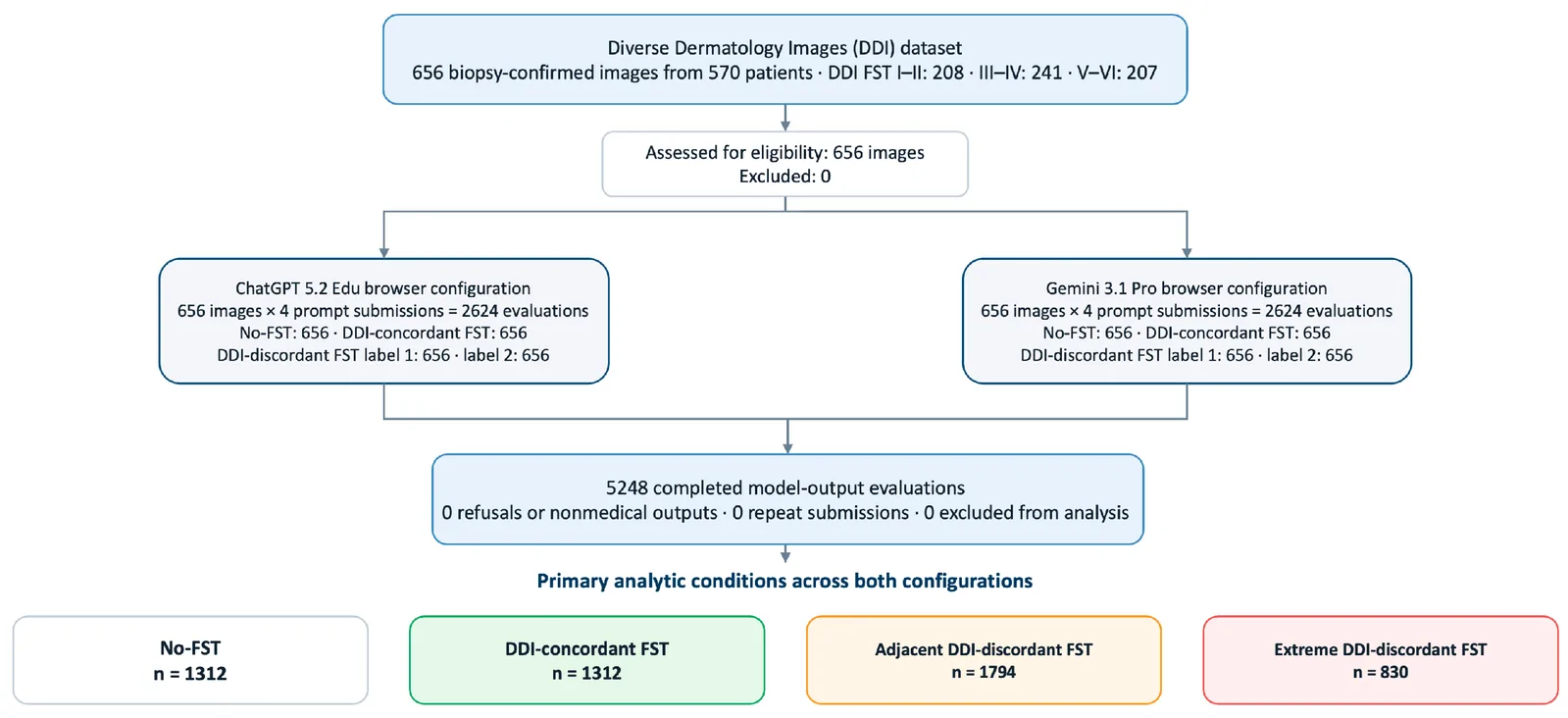
Study flow diagram. All 656 biopsy-confirmed photographs underwent four prompt submissions per model: no-FST, DDI-concordant FST, and two DDI-discordant FST labels, yielding 5248 evaluations. The two DDI-discordant submissions per image were recoded as adjacent or extreme discordance relative to the DDI-assigned FST group. FST III–IV images contributed two adjacent observations and no extreme observation; FST I–II and V–VI images each contributed one adjacent and one extreme observation. No images were excluded. DDI, Diverse Dermatology Images; FST, Fitzpatrick skin type.

Each image was evaluated under four prompt conditions per model: (1) no-FST, denoting a diagnostic text prompt without FST metadata; (2) DDI-concordant FST, with the DDI-assigned FST group included; (3) DDI-discordant FST prompt 1, with one of the other grouped FST labels provided; and (4) DDI-discordant FST prompt 2, with the remaining grouped FST label provided. This yielded 2624 evaluations per model and 5248 total across both models (Figure 1). Each image served as its own control, controlling for lesion type, image quality, and diagnosis across prompt conditions.

DDI-discordant FST prompts were classified by degree of discordance from the DDI-assigned FST group. An adjacent DDI-discordant prompt denoted one grouped FST category of discordance (for example, a DDI FST I–II image labeled as FST III–IV). An extreme DDI-discordant prompt denoted maximal discordance, two grouped categories apart (for example, a DDI FST I–II image labeled as FST V–VI, or vice versa). Because both nonconcordant labels for DDI FST III–IV images represent adjacent discordance by definition, FST III–IV images did not contribute to the extreme discordance comparison.

The verbatim prompt wording was as follows. All conditions began: “You are a dermatology diagnostic assistant. Your task is to analyze the provided clinical image and generate a ranked differential diagnosis. Do not include management or treatment recommendations.” The no-FST condition then continued: “Please review the attached clinical photograph and provide a differential diagnosis for the skin lesion. List the top 3 most likely diagnoses in order of probability. Do not include explanations.” For the FST-labeled conditions, the sentence “This patient has Fitzpatrick skin type [INSERT TYPE].” was inserted before the same diagnostic request; [INSERT TYPE] was populated with the DDI-concordant or DDI-discordant grouped label. The full prompt templates are provided in Document S1.

### 2.4. Data Collection

Case IDs were randomly assigned prior to data collection among three study team members (MB, JT, TW), each contributing responses for approximately one-third of the image set. Each data collector applied all four prompt conditions to their assigned images using a standardized, prespecified procedure. There was no prespecified or counterbalanced order of data input of the four prompt conditions, and submission order was not recorded. Data quality control was performed centrally by JM, including verification of output formatting and removal of non-diagnostic framing before or after the ranked differential (preambles, caveats, disclaimers, and similar). The ranked differential itself was not altered. Raw pre-trimming responses were not retained. All 5248 submissions returned a ranked differential; no refusals, non-medical responses, or repeat submissions occurred.

### 2.5. Outcome Measures and Scoring

The primary outcome was an ordinal diagnostic accuracy score from 0 to 3, assigned relative to histopathologic ground truth: 3 if the correct diagnosis was ranked first among the model’s top three differential diagnoses, 2 if ranked second, 1 if ranked third, and 0 if absent from the top three entirely.

Scoring was performed centrally by a single reviewer (MB), blinded to the prompt condition, using a prespecified scoring ontology (Document S2). Processed ranked differential lists were placed in a randomized Excel scoring worksheet without prompt condition labels, and the reviewer compared each ranked list with the corresponding DDI diagnosis. Diagnoses were accepted as correct if they matched the histopathologic ground truth at the disease-entity level or fell within a predefined clinically equivalent family. Six diagnostic families were treated as collapsed groups: melanoma, squamous cell carcinoma, basal cell carcinoma, benign melanocytic lesions, seborrheic keratosis, and verruca. Any prediction within the same family as the ground truth was accepted as correct regardless of subtype. The full collapsing rules and entity-level equivalences are provided in Appendix A. The scoring proceeded from rank 1 to rank 3, stopping at the first match. Ambiguous cases were adjudicated by a board-certified dermatologist (NJ). Outputs that declined to diagnose or returned non-medical responses were prespecified to score 0; none occurred. Single-reviewer scoring ensured uniform ontology application but precluded inter-rater reliability estimation.

The prespecified exploratory safety outcome was confirmed malignant diagnosis omission (“lethal miss”), defined as the proportion of malignant images for which the biopsy-confirmed diagnosis or accepted ontology equivalent was entirely absent from the model’s top three differential diagnoses (score = 0), reported by model and prompt condition. This outcome is not equivalent to a binary false-negative classification for malignancy because a response could omit the confirmed malignant diagnosis while listing a different malignant diagnosis.

### 2.6. Statistical Analysis

The primary analysis used a cumulative link mixed-effects model (CLMM) with a logit link to model the ordinal diagnostic accuracy score. Model identity (ChatGPT 5.2 vs. Gemini 3.1 Pro) and prompt condition (no-FST, DDI-concordant FST, adjacent DDI-discordant FST, and extreme DDI-discordant FST) were included as fixed effects, with a random intercept for image to account for within-image correlation across repeated prompt submissions. The proportional odds assumption was assessed by likelihood ratio comparison of an additive cumulative link model with a model allowing nominal condition effects; a partial proportional odds or nominal model was prespecified if a significant violation was detected. A model-by-condition interaction was evaluated by likelihood ratio comparison of additive and interaction CLMMs before primary inference; in the absence of a significant interaction, the additive model was used.

Four prespecified hypotheses were evaluated: the overall model effect (Gemini vs. ChatGPT) and three prompt condition comparisons against the no-FST reference (DDI-concordant FST, adjacent DDI-discordant FST, and extreme DDI-discordant FST). ChatGPT and the no-FST condition served as the reference categories. The *p* values for these four primary tests were adjusted together using the Benjamini–Hochberg false discovery rate procedure at an expected FDR of 10%. The results are reported as odds ratios (ORs) with 95% confidence intervals (CIs); an OR greater than 1 indicates greater odds of receiving a higher diagnostic accuracy score.

Two prespecified exploratory analyses were conducted. First, malignant lethal misses were summarized separately by model and prompt condition. For descriptive aggregation of the adjacent DDI-discordant condition, each FST I–II or FST V–VI image contributed its single adjacent prompt outcome; because both DDI-discordant prompts were adjacent for FST III–IV images, their two binary lethal miss indicators were averaged within each image to preserve equal image-level weighting. Formal paired testing was limited, as prespecified, to the comparison of no-FST and extreme DDI-discordant prompting, separately for each model, among the 97 malignant images for which an extreme discordance condition was available (FST I–II and V–VI only). FST III–IV malignant images were excluded because neither nonconcordant label represented extreme discordance. This comparison used the continuity-corrected McNemar test, with the paired OR calculated as the ratio of discordant images changing from non-miss to lethal miss to those changing from lethal miss to non-miss. The McNemar *p* values were nominal. Second, baseline FST accuracy disparities in the no-FST condition were assessed separately by model and malignancy status using the Kruskal–Wallis test across the three FST groups. Significant omnibus results were followed by the two-sided pairwise Mann–Whitney U tests with Bonferroni correction across the three comparisons, with rank-biserial correlations reported as effect sizes. All exploratory findings were considered hypothesis-generating.

Because the recoded conditions had unequal FST group contributions, a post hoc equal-image-weighted sensitivity CLMM was conducted. Each of the two adjacent DDI-discordant observations from an FST III–IV image received a weight of 0.5, and all other observations received a weight of 1, so that each image contributed the same total weight to the adjacent condition. All 5248 records were retained (total case weight, 4766). DDI-assigned FST group was included as an additional fixed effect; the model, prompt condition, and the image random intercept were otherwise specified as in the primary model. The seven-point adaptive Gauss–Hermite quadrature was used for the reporting fit, and a five-point fit was used as a numerical stability check. This post hoc analysis did not replace the prespecified primary model.

As a post hoc descriptive analysis, top-1 diagnostic accuracy was defined as the proportion of evaluations for which the biopsy-confirmed diagnosis or accepted ontology equivalent was ranked first (score = 3), and top-3 diagnostic inclusion accuracy was defined as the proportion for which it appeared anywhere in the ranked differential (score > 0). For FST III–IV images, the two adjacent DDI-discordant binary indicators were averaged within image so that each image contributed equal total weight. Extreme DDI-discordant estimates were restricted to the 415 images assigned to DDI FST I–II or V–VI. No additional hypothesis testing was performed for these descriptive outcomes.

Additional post hoc descriptive analyses were conducted to further evaluate diagnostic performance. For binary malignancy detection, a response was positive if any of the top three diagnoses matched an explicit malignant entity or family term (melanoma, basal cell carcinoma, squamous cell carcinoma, other carcinoma, metastatic disease, lymphoma/leukemia, sarcoma, or another explicitly malignant tumor) and negative otherwise. Premalignant or benign terms, including actinic keratosis, dysplastic nevus, and Spitz/Reed nevus, were negative unless an explicit malignancy was also listed. Using the DDI malignancy field as the reference, sensitivity, specificity, positive predictive value (PPV), negative predictive value (NPV), F1 score, accuracy, and underlying true-positive (TP), false-positive (FP), true-negative (TN), and false-negative (FN) counts were calculated for each model and each of the four original prompt submissions. Wilson 95% confidence intervals were calculated for all proportions except F1. For the top-1 error-pattern analysis, one fixed prediction crosswalk was constructed across both configurations and all four original prompt submissions before matrix tabulation. A normalized diagnosis string was linked to a canonical category when the original scorer had accepted that string as a match at any rank; all links were unique. The remaining strings were mapped through the ontology family scope, exact entity labels, and documented equivalences only. For strings not established by a prior score match, the principal diagnosis before any parenthetical descriptor was used. Predictions that were uncertain, multi-category, morphologic-only, or outside the ontology were assigned to Other/unmapped. Audit checks confirmed that every score-3 output fell on its ground-truth diagonal, every off-diagonal entry had a score below 3, and each matrix contained 656 observations. Complete count and row-normalized matrices were generated for both configurations under all four original prompt submissions. The original submissions were used because each contributes one response per image, whereas the recoded adjacent and extreme conditions have unequal FST group contributions. For readability, the main-text figure condenses the no-FST matrices by displaying the 12 most prevalent ground-truth canonical categories and grouping the remaining mapped categories as Other categories; the complete unaggregated matrices are provided in Workbook S1. The crosswalk was applied after scoring and left the prespecified ordinal scores unchanged; no additional hypothesis testing was performed. The prediction crosswalk and complete mapping audit are included with the deposited analysis files.

## 3. Results

### 3.1. Primary Diagnostic Accuracy and Prompting Effects

Across 5248 prompt-image evaluations, Gemini 3.1 Pro had substantially greater ordinal diagnostic accuracy than ChatGPT 5.2 (OR 2.32; 95% CI 2.01–2.67; FDR-adjusted *p* < 0.001). No FST prompt condition significantly improved diagnostic accuracy relative to no-FST prompting in the primary CLMM. The proportional odds assumption was not violated (*p* = 0.586), and the model-by-condition interaction was not significant (*p* = 0.889), supporting use of the additive model for primary inference. After FDR correction, no FST prompt condition significantly differed from no-FST prompting (Table 1). DDI-concordant FST prompting produced a modest, non-significant directional increase over no-FST (OR 1.16; 95% CI 0.95–1.40; adjusted *p* = 0.283). Adjacent DDI-discordant prompting showed negligible change (OR 0.99; 95% CI 0.82–1.18; adjusted *p* = 0.893). Extreme DDI-discordant prompting showed a directional decrease that did not reach the corrected threshold (OR 0.88; 95% CI 0.70–1.12; adjusted *p* = 0.398).

In the post hoc equal-image-weighted sensitivity analysis adjusting for DDI-assigned FST group, the Gemini configuration retained higher odds of a higher diagnostic score than the ChatGPT configuration (OR 2.25; 95% CI 1.94–2.61; nominal *p* < 0.001). The prompt condition estimates remained close to the primary estimates: DDI-concordant versus no-FST OR 1.15 (95% CI 0.95–1.40; *p* = 0.145), adjacent DDI-discordant versus no-FST OR 0.98 (95% CI 0.81–1.19; *p* = 0.857), and extreme DDI-discordant versus no-FST OR 0.93 (95% CI 0.73–1.17; *p* = 0.526). Thus, equal image weighting and adjustment for DDI-assigned FST group did not alter the qualitative conclusion (Table S1).

The post hoc descriptive top-1 and top-3 diagnostic accuracy estimates are reported in Table S2. In the no-FST condition, top-1 and top-3 accuracy were 23.0% and 45.4%, respectively, for ChatGPT and 31.6% and 53.5% for Gemini. Under DDI-concordant FST prompting, the corresponding values were 25.3% and 46.8% for ChatGPT and 32.6% and 54.4% for Gemini; under adjacent DDI-discordant prompting, they were 22.7% and 46.0% for ChatGPT and 31.9% and 52.6% for Gemini. In the 415-image outer-group subset eligible for extreme discordance, top-1 and top-3 accuracy were 17.3% and 38.8% for ChatGPT and 25.1% and 45.5% for Gemini. These estimates were descriptive and were not subjected to additional hypothesis testing.

### 3.2. Baseline Diagnostic Disparities by FST Group

Evaluation of the unprimed (no-FST) condition revealed significant baseline disparities across DDI-assigned FST groups, stratified by malignancy status. For ChatGPT 5.2, among malignant images, accuracy for FST V–VI was significantly lower than for both FST I–II (mean score 0.69 vs. 1.37; Bonferroni *p* = 0.027) and FST III–IV (mean score 0.69 vs. 1.45; Bonferroni *p* = 0.005). Among benign images, ChatGPT performed significantly better on FST III–IV than FST I–II (mean score 1.22 vs. 0.78; Bonferroni *p* = 0.001).

**Figure 2:**
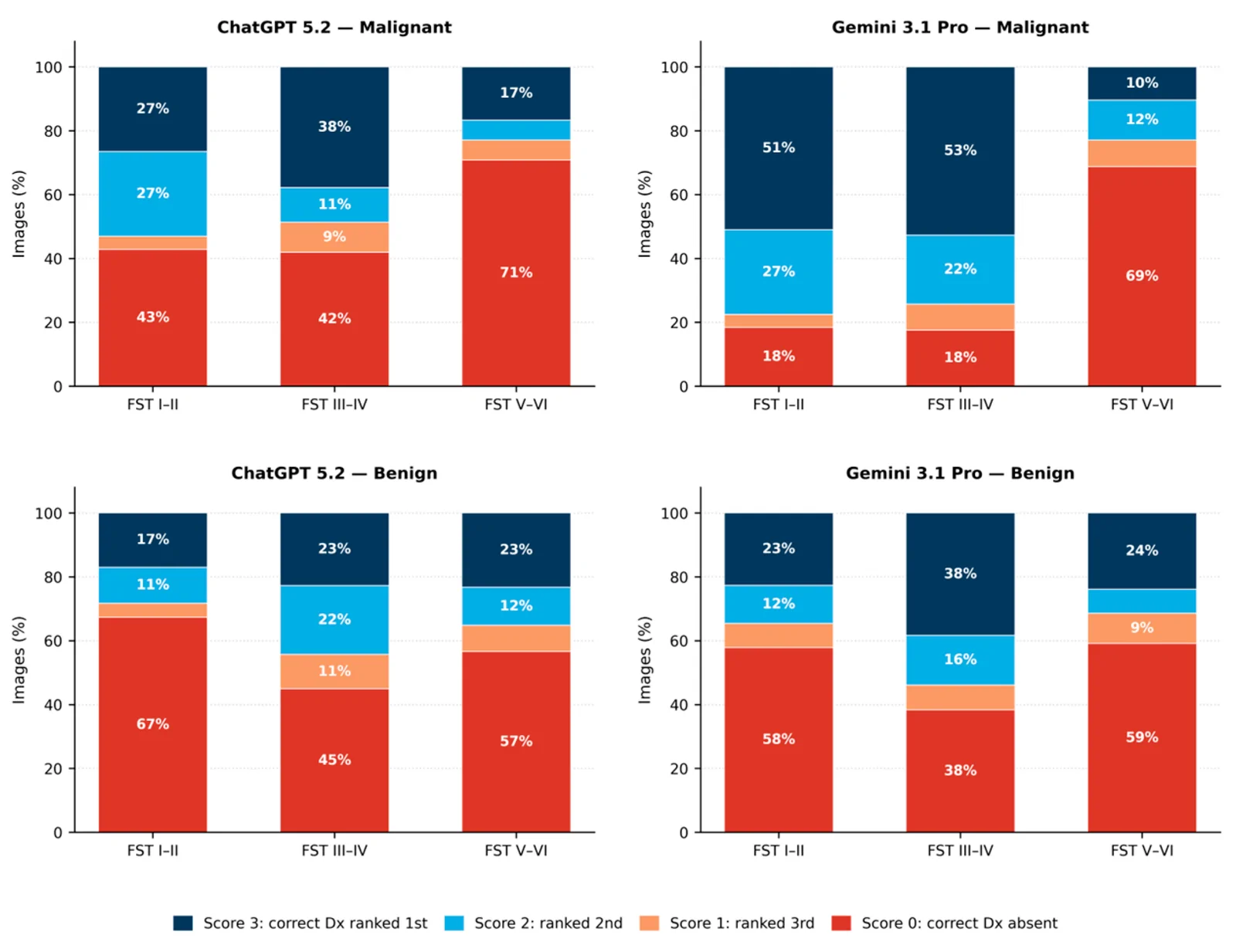
Ordinal diagnostic accuracy score distributions by DDI-assigned Fitzpatrick skin type group in the no-FST condition. The panels show the percentage of images receiving each score (0–3) for ChatGPT 5.2 (**left**) and Gemini 3.1 Pro (**right**), stratified by malignancy status (**top**: malignant; **bottom**: benign). Score 3 indicates that the correct diagnosis was ranked first among the top three differential diagnoses; score 0 indicates that it was absent from the top three. Dx, diagnosis; FST, Fitzpatrick skin type.

Gemini 3.1 Pro exhibited a more pronounced disparity among malignant images. Accuracy for DDI FST V–VI was substantially lower than for both DDI FST I–II (mean score 0.65 vs. 2.10; rank-biserial *r* = −0.595; Bonferroni *p* < 0.001) and DDI FST III–IV (mean score 0.65 vs. 2.09; rank-biserial *r* = −0.599; Bonferroni *p* < 0.001) (Figure 2). The full stratified results are available in Table 2.

### 3.3. Confirmed Malignant Diagnosis Omission (“Lethal Miss”) Analysis

**Figure 3:**
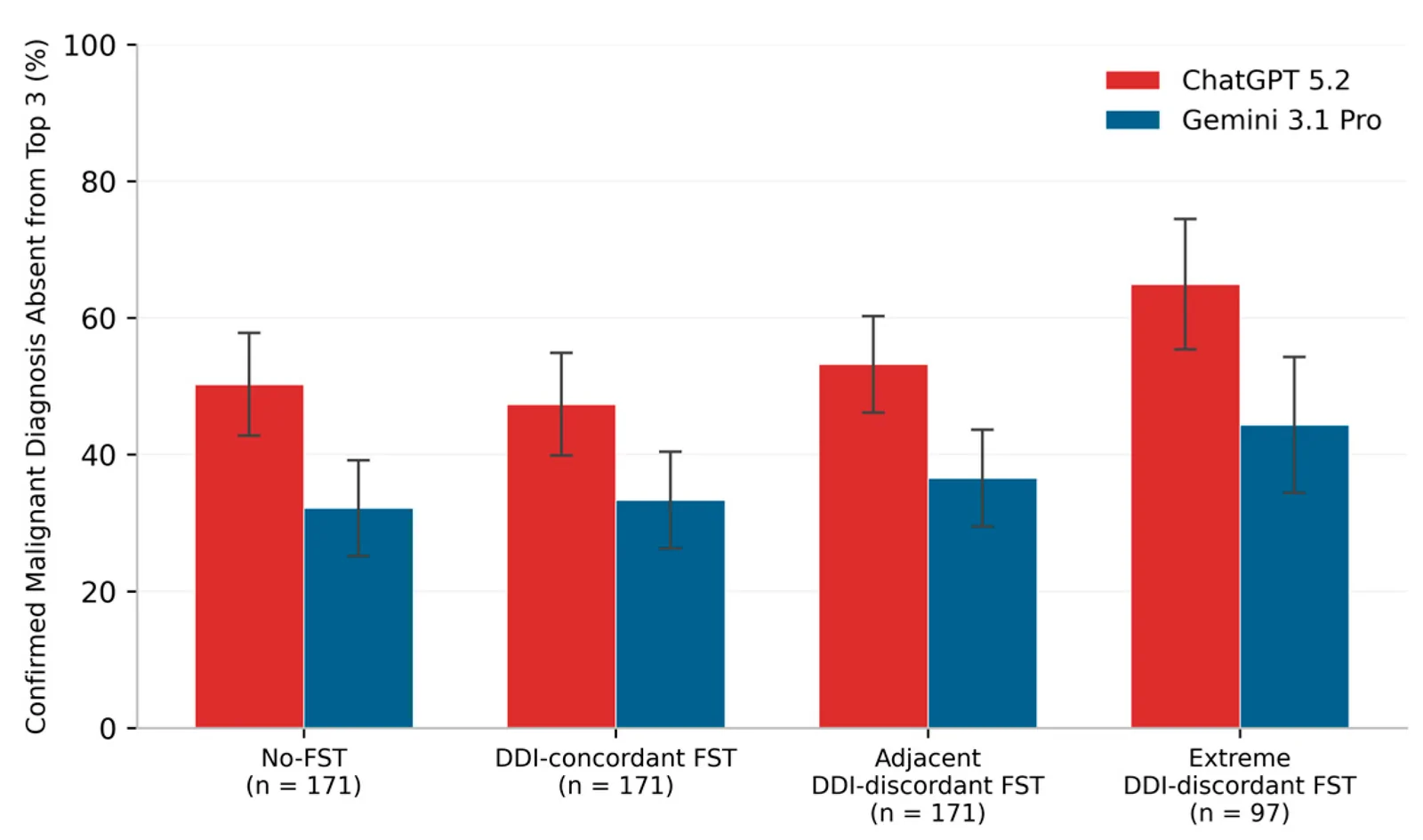
Confirmed malignant diagnosis omission (“lethal miss”) rates by model and prompt condition. A lethal miss denotes absence of the biopsy-confirmed diagnosis or accepted ontology equivalent from the top three differential diagnoses and should not be interpreted as a binary failure to identify any malignancy because the differential could contain another malignant diagnosis. Error bars indicate 95% confidence intervals (normal approximation). Note: Extreme DDI-discordant analysis was restricted to FST I–II and V–VI images (*n* = 97). For the adjacent DDI-discordant condition, the two outcomes for FST III–IV images were averaged to preserve equal image-level weighting.

Among 171 malignant images, no-FST confirmed malignant diagnosis omission (“lethal miss”) rates were 86/171 (50.3%) for ChatGPT 5.2 and 55/171 (32.2%) for Gemini 3.1 Pro. The corresponding rates under DDI-concordant FST prompting were 81/171 (47.4%) for ChatGPT and 57/171 (33.3%) for Gemini. To maintain image-level weighting, outcomes for FST III–IV images under adjacent DDI-discordant prompting were averaged within each image before aggregation, yielding an averaged image-level miss burden of 91.0 (53.2%) for ChatGPT and 62.5 (36.5%) for Gemini. With extreme DDI-discordant FST prompting, among the 97 eligible malignant images (FST I–II and V–VI only), the rates were 63/97 (64.9%) for ChatGPT and 43/97 (44.3%) for Gemini. Within the same 97-image extreme discordance subset used for paired testing, no-FST lethal misses were 55/97 (56.7%) for ChatGPT and 42/97 (43.3%) for Gemini. In paired testing against the no-FST baseline, extreme DDI-discordant prompting was associated with more ChatGPT transitions to lethal miss: 10 images shifted from non-miss (score > 0) to lethal miss (score = 0), and two shifted from lethal miss to non-miss (paired OR 5.0; continuity-corrected nominal *p* = 0.043). Gemini showed no significant paired shift: six images shifted from non-miss to lethal miss, and five shifted from lethal miss to non-miss (paired OR 1.2; *p* = 1.00). The full lethal miss data by condition are provided in Figure 3 and Table 3.

### 3.4. Post Hoc Binary Malignancy Detection and Top-1 Confusion Matrices

**Figure 4:**
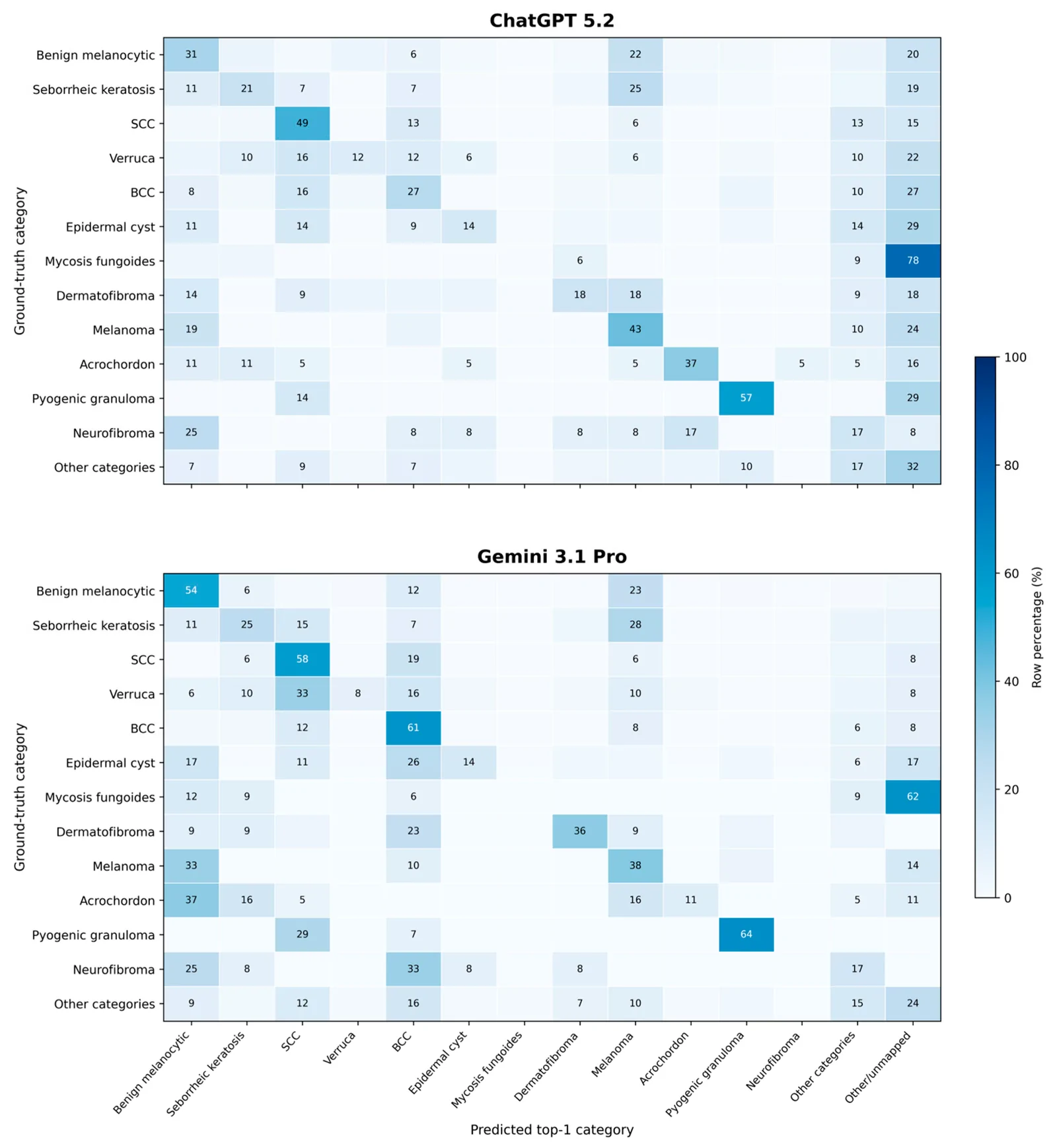
Condensed row-normalized top-1 confusion matrices under no-FST prompting for ChatGPT 5.2 (**top**) and Gemini 3.1 Pro (**bottom**). The 12 most prevalent ground-truth categories are shown; the remaining mapped categories are grouped as Other categories and unresolved predictions as Other/unmapped. Because multiple uncommon ground-truth and predicted categories are collapsed, the Other categories × Other categories cell should not be interpreted as category-level correct classification. Values below 5% are not labeled. Complete unaggregated matrices for all four original prompt submissions are provided in Workbook S1.

The post hoc binary malignancy detection metrics are reported in Table S3. Under no-FST prompting, the ChatGPT sensitivity, specificity, positive predictive value, negative predictive value, F1 score, and accuracy were 67.3%, 42.5%, 29.2%, 78.6%, 40.7%, and 48.9%, respectively; the corresponding Gemini values were 81.3%, 22.9%, 27.1%, 77.6%, 40.6%, and 38.1%. Across all four original submissions, specificity remained low (ChatGPT, 32.2–49.3%; Gemini, 19.0–23.5%), indicating frequent malignancy overcalling. These metrics assess whether any malignancy was raised and are distinct from confirmed malignant diagnosis omission. In the no-FST top-1 error-pattern analysis, 489 of 656 ChatGPT outputs (74.5%) and 576 of 656 Gemini outputs (87.8%) received a canonical mapping; the remaining predictions were retained as Other/unmapped. All links were unique and consistent with the prespecified ordinal scores. The condensed row-normalized no-FST confusion matrices are shown in Figure 4. Frequent mapped errors included benign melanocytic lesions and seborrheic keratoses classified as melanoma, verrucae classified as squamous cell carcinoma, and cross-classification between basal and squamous cell carcinoma. The complete unaggregated count and row-normalized matrices for both configurations under all four original prompt submissions are provided in Workbook S1.

## 4. Discussion

FST prompting did not significantly improve diagnostic accuracy for either model tested, and extreme DDI-discordant FST labels were associated with more ChatGPT malignant lethal misses in the prespecified exploratory paired analysis. Clinicians should not assume that specifying a patient’s FST improves LLM diagnostic accuracy or reduces malignant lethal miss rates, particularly in the model–interface and dataset configurations evaluated here. For Gemini, no-FST accuracy was lower for malignant DDI FST V–VI images than for malignant DDI FST I–II and III–IV images, indicating a separate baseline performance disparity in this dataset.

This experiment evaluated a single-image differential diagnosis task, not a complete clinical encounter. Each model received one static photograph and a standardized prompt; there was no lesion history, symptoms, temporal evolution, palpation, dermoscopic findings, full-body examination, comorbidities, medication history, or opportunity to ask clarifying questions. Biopsy confirmation strengthened the reference diagnosis but did not make the model input representative of real clinical reasoning. Performance on this curated image benchmark should not be extrapolated to clinical utility, patient outcomes, or performance during an actual encounter.

The confirmed diagnosis omission outcome likewise should not be interpreted as a binary false-negative classification for malignancy: a response could omit the biopsy-confirmed malignancy while listing a different malignant diagnosis. The evaluated general-purpose browser configurations are not appropriate for autonomous dermatologic diagnosis or as replacements for dermatologists. In a 1000-case dermatology triage evaluation with 50 repeated trials, specialized neural networks significantly outperformed GPT-4o on most pairwise comparisons [29]. In a prospective two-center study, dermatologists who incorporated the output of a market-approved convolutional neural network improved mean sensitivity, specificity, accuracy, and area under the receiver operating characteristic curve compared with their unaided assessments [30]. For a defined classification task, a purpose-built system trained and independently validated for its intended input and population is a more meaningful point of comparison than a general-purpose conversational model. Because the present study did not directly compare the evaluated configurations with a task-specific model, it does not establish that all multimodal LLM architectures are fundamentally unsuitable for dermatology. Task-specific systems also require independent prospective validation, subgroup assessment, and clinician oversight before deployment.

In the post hoc binary analysis, sensitivity exceeded specificity, particularly for Gemini, while low specificity and PPV indicated substantial malignancy overcalling. The no-FST confusion matrices further identified recurrent clinically important errors, including benign melanocytic lesions and seborrheic keratoses classified as melanoma, verrucae classified as squamous cell carcinoma, and cross-classification between basal and squamous cell carcinoma. Uncertain or out-of-ontology predictions were retained as Other/unmapped rather than forced into a diagnostic category. Similar patterns of low specificity, hallucinated findings, and inconsistent accuracy have been reported across dental radiography, general radiology, fracture detection, and brain-metastasis MRI [12,13,14,15]. An illustrative five-model head CT study likewise documented one fundamental diagnostic error and clinically meaningful intermodel disagreement and cautioned against autonomous interpretation. While other investigators have reached stronger conclusions regarding their unsuitability for autonomous use [16], that single-case study provides conceptual rather than population-level evidence. Although selected curated, text-rich benchmarks have reported stronger performance, the broader evidence remains heterogeneous and does not establish autonomous clinical reliability [11]. Together, these findings support clinician oversight rather than standalone diagnostic use.

The absence of improvement with DDI-concordant FST prompting may reflect a mismatch between how skin tone is used clinically and how multimodal LLMs process contextual text. While lay patients are unlikely to use formal FST terminology in prompts, this study used FST because it is the predominant skin tone metadata structure in dermatology AI datasets, making it a natural test of how these models handle demographic context. A clinician uses skin tone to shift prior probabilities for diagnoses that present differently or occur at different rates across populations. When a multimodal LLM receives an FST label, it processes a categorical text token that may interact with the visual signal. One possibility is that the label is insufficiently informative or competes with visual evidence when it is discordant; the present study did not directly evaluate model-internal mechanisms.

ChatGPT’s paired OR of 5.0 was observed in the exploratory extreme discordance analysis and requires replication. Gemini did not show a statistically significant paired shift. Separately, Gemini showed lower malignant diagnostic accuracy for DDI FST V–VI images than for DDI FST I–II and III–IV images in the no-FST condition. This no-FST disparity is consistent with the prior literature linking dermatology AI inequities to dataset composition, representation gaps, and incomplete skin tone metadata [6,23,31,32]. The present study did not formally test whether prompt condition modified this disparity. Future studies should evaluate prompt-by-FST interactions and identify the diagnoses contributing to subgroup differences using richer demographic metadata. FST was designed to capture UV response and photosensitivity rather than to measure skin tone, race, ethnicity, or ancestry, and these constructs are not interchangeable [20,24,25]. The DDI dataset did not include sex or gender metadata, so those comparisons were not possible here. Prior work has shown that gender-coded names shift ChatGPT-generated language [33] and that demographic stratification reveals subgroup-specific patterns in computational text analyses [34]; similar analyses in multimodal dermatology datasets with richer demographic metadata would be a productive next step.

Given that FST and skin tone assessment are not standardized and may be inferred or inconsistently documented [20,21,22,23], the present results do not support adding FST labels to improve diagnostic accuracy in the evaluated configurations. The pattern of findings argues for caution in incorporating FST or similar skin tone descriptors into LLM diagnostic prompts until their effects are better characterized. In this dataset and these model–interface configurations, FST prompting did not significantly change diagnostic accuracy in most comparisons and was associated with more ChatGPT lethal misses in the prespecified extreme discordance safety analysis.

Limitations of this study include restriction to a single dataset from a single academic center, which may limit generalizability. The DDI data available for this study lacked race, ethnicity, sex, gender, and anatomic location metadata, constraining bias analyses to FST-level comparisons. Patient-level linkage identifiers were unavailable in the analytic files, so possible correlation among multiple images from the same patient could not be modeled. DDI-assigned FST was not independently verified with colorimetry or additional dermatologist review, and per-image assignment uncertainty was unavailable. The grouped FST categories precluded examination of within-pair differences. FST III–IV images contributed two adjacent DDI-discordant observations and no extreme DDI-discordant observation, so the primary adjacent estimate drew on unequal image contributions while the extreme estimate covered only the outer FST groups; a post hoc equal-image-weighted sensitivity analysis adjusting for DDI-assigned FST group produced similar prompt condition estimates. Submission order across the four conditions was not prescribed or recorded; each submission did occur in a fresh temporary conversation without prior visible context. Pre-trimming outputs were not retained. The scoring reviewer was blinded to prompt condition, but single-reviewer scoring precluded inter-rater reliability estimation. The single-query design does not capture stochastic output variability. Post-upload resolution, compression, hidden instructions, safety settings, and backend routing were not observable through either browser interface. The confusion matrices are post hoc reductions of open-ended ranked differentials. Although the score-anchored crosswalk preserved consistency with the prespecified scoring, 10.7–30.0% of top-ranked outputs across the eight model-condition matrices remained Other/unmapped. The condensed main-text matrices aggregate less prevalent categories; the complete unaggregated matrices and the mapping audit are provided in the supplementary and deposited materials. The static-photograph task also did not reproduce the information or interaction available during a complete clinical encounter. All reported estimates are aggregate properties of this dataset and these configurations; they do not reflect expected accuracy for any individual patient. Establishing clinical utility would require prospective evaluation in the intended population, calibration assessment, integration with full clinical context, and clinician oversight. The ChatGPT lethal miss finding rests on 97 eligible malignant images and warrants replication in larger datasets before broader conclusions are drawn.

## 5. Conclusions

FST prompting did not significantly improve diagnostic accuracy in either evaluated configuration. Extreme DDI-discordant FST metadata was associated with more ChatGPT lethal misses in the exploratory paired analysis, while Gemini showed a no-FST FST-stratified disparity. The post hoc binary analysis additionally showed low specificity and positive predictive value, indicating frequent malignancy overcalling. Neither configuration demonstrated reliability sufficient for autonomous dermatologic diagnosis. Together with recent cross-domain evidence, these findings are consistent with an emerging consensus that current general-purpose multimodal LLMs are not ready for autonomous clinical image interpretation and should not replace clinicians or validated task-specific diagnostic systems. Findings apply to the evaluated dataset and browser configurations and do not establish that FST metadata is universally harmful or that one underlying architecture is intrinsically superior.

## Figures and Tables

**Table 1:** Primary CLMM Results (no-FST/ChatGPT reference).

Comparison	OR	95% CI	Nominal *p*	FDR-Adj. *p* (BH)
Gemini vs. ChatGPT	2.32	2.01–2.67	<0.001	<0.001
DDI-Concordant FST vs. No-FST	1.16	0.95–1.40	0.141	0.283
Adjacent DDI-Discordant vs. No-FST	0.99	0.82–1.18	0.893	0.893
Extreme DDI-Discordant vs. No-FST	0.88	0.70–1.12	0.299	0.398

BH, Benjamini–Hochberg; CI, confidence interval; CLMM, cumulative link mixed model; LRT, likelihood-ratio test; FDR, false discovery rate; FST, Fitzpatrick skin type; OR, odds ratio. Note: Model-by-condition interaction *p* = 0.889; proportional odds LRT *p* = 0.586; additive model used for primary inference. OR > 1 indicates higher odds of a higher accuracy category.

**Table 2:** Baseline DDI-assigned FST accuracy disparities (no-FST condition).

Model	Stratum	Comparison	*n*_1_	Mean_1_	*n*_2_	Mean_2_	KW *p*	Rank-Biserial *r*	Unadjusted *p*	Bonferroni *p*
ChatGPT 5.2	Malignant	FST I–II vs. III–IV	49	1.37	74	1.45	0.005	0.051	0.613	1.000
ChatGPT 5.2	Malignant	FST III–IV vs. V–VI	74	1.45	48	0.69	0.005	−0.304	0.002	0.005
ChatGPT 5.2	Malignant	FST I–II vs. V–VI	49	1.37	48	0.69	0.005	−0.276	0.009	0.027
ChatGPT 5.2	Benign	FST I–II vs. III–IV	159	0.78	167	1.22	0.002	0.202	<0.001	0.001
ChatGPT 5.2	Benign	FST III–IV vs. V–VI	167	1.22	159	1.02	0.002	−0.092	0.119	0.357
ChatGPT 5.2	Benign	FST I–II vs. V–VI	159	0.78	159	1.02	0.002	0.105	0.061	0.184
Gemini 3.1 Pro	Malignant	FST I–II vs. III–IV	49	2.10	74	2.09	<0.001	0.005	0.962	1.000
Gemini 3.1 Pro	Malignant	FST III–IV vs. V–VI	74	2.09	48	0.65	<0.001	−0.599	<0.001	<0.001
Gemini 3.1 Pro	Malignant	FST I–II vs. V–VI	49	2.10	48	0.65	<0.001	−0.595	<0.001	<0.001
Gemini 3.1 Pro	Benign	FST I–II vs. III–IV	159	0.99	167	1.54	<0.001	0.220	<0.001	<0.001
Gemini 3.1 Pro	Benign	FST III–IV vs. V–VI	167	1.54	159	0.96	<0.001	−0.226	<0.001	<0.001
Gemini 3.1 Pro	Benign	FST I–II vs. V–VI	159	0.99	159	0.96	<0.001	−0.010	0.862	1.000

Note: Bonferroni correction for 3 pairwise comparisons per stratum (α = 0.0167). KW *p* shown per stratum (shared across pairwise comparisons within that stratum).

**Table 3:** Confirmed malignant diagnosis omission (“Lethal Miss”) by Condition.

Model	Prompt Condition	Eligible Malignant Images (*n*)	Lethal Misses/Image-Level Miss Burden	Lethal Miss Rate
ChatGPT 5.2	No-FST	171	86	50.3%
ChatGPT 5.2	DDI-Concordant FST	171	81	47.4%
ChatGPT 5.2	Adjacent DDI-Discordant FST	171	91.0	53.2%
ChatGPT 5.2	Extreme DDI-Discordant FST	97	63	64.9%
Gemini 3.1 Pro	No-FST	171	55	32.2%
Gemini 3.1 Pro	DDI-Concordant FST	171	57	33.3%
Gemini 3.1 Pro	Adjacent DDI-Discordant FST	171	62.5	36.5%
Gemini 3.1 Pro	Extreme DDI-Discordant FST	97	43	44.3%

Note: Extreme discordance analysis was restricted to FST I–II and V–VI malignant images (*n* = 97). For adjacent discordance, the two prompt outcomes for FST III–IV images were averaged within image before aggregation to maintain image-level weighting.

## Data Availability

The dataset supporting the results of this study, including processed model outputs, diagnostic scores, the analysis notebook, prompt templates, predefined scoring ontology, study protocol, binary malignancy results, prediction crosswalk, mapping audit, and complete confusion matrices, is openly available on Mendeley Data at https://doi.org/10.17632/z4dw2djvkk.

## Supplementary Materials

The following supporting information can be downloaded at: https://www.mdpi.com/article/10.3390/bioengineering13080932/s1. Document S1: Prompt templates; Document S2: Predefined scoring ontology; Table S1: Post hoc equal-image-weighted sensitivity CLMM; Table S2: Post hoc descriptive top-1 and top-3 diagnostic accuracy; Table S3: Post hoc binary malignancy detection; Workbook S1: Complete post hoc top-1 confusion matrices.

## Abbreviations

The following abbreviations are used in this manuscript:

AI	Artificial Intelligence
BCC	Basal Cell Carcinoma
BH	Benjamini–Hochberg
CI	Confidence Interval
CLMM	Cumulative Link Mixed Model
DDI	Diverse Dermatology Images
FDR	False Discovery Rate
FST	Fitzpatrick Skin Type
KW	Kruskal–Wallis
LLM	Large Language Model
LRT	Likelihood Ratio Test
NPV	Negative Predictive Value
OR	Odds Ratio
PPV	Positive Predictive Value
SCC	Squamous Cell Carcinoma

## Appendix A. The Scoring Ontology: Collapsing Rules and Adjudication Notes

The prespecified scoring ontology mapped all 78 histopathologic ground-truth diagnostic entries from the DDI dataset to a canonical category, each designated as either family-level (collapsed) or exact entity-level. The complete entry-by-entry mapping is provided in Document S2. The rules governing family-level matching, entity-level equivalences, and adjudication decisions applied during scoring are described below.

General scoring rules. Only the top three ranked predictions were evaluated. Scoring stopped at the first prediction matching the ground-truth canonical category (rank 1 = 3 points; rank 2 = 2 points; rank 3 = 1 point; absent from top 3 = 0 points). A prediction was correct if it mapped to the same canonical category as the ground truth and incorrect otherwise. Diagnoses were not considered correct solely on the basis of broad clinical class membership (e.g., inflammatory, infectious, or vascular). Collapsing across malignant and benign entities was not permitted. Subtype specificity did not override rank position.

Collapsed subtype families. Six diagnostic families were treated as collapsed groups, meaning any prediction within the same family as the ground truth was accepted as correct regardless of subtype: “melanoma_family” (melanoma, melanoma in situ, acral lentiginous melanoma, nodular melanoma); “scc_family” (squamous cell carcinoma, SCC in situ, keratoacanthoma-type SCC); “bcc_family” (basal cell carcinoma, nodular BCC, superficial BCC); “benign_melanocytic_family” (melanocytic nevi, blue nevus, dysplastic nevus, atypical spindle cell nevus of Reed, pigmented spindle cell nevus of Reed, acral melanotic macule); “seborrheic_keratosis_family” (seborrheic keratosis, irritated seborrheic keratosis, benign keratosis, inverted follicular keratosis, lichenoid keratosis, dermatosis papulosa nigra); and “verruca_family” (verruca vulgaris, wart, verruca plana, verruca plantaris, verruca filiformis). Of the 78 entries, 23 were assigned to one of these six families; the remaining 55 required exact entity-level matching.

Entity-level equivalences. The following equivalences were applied outside the collapsed families. Seborrheic dermatitis, nummular dermatitis, and nummular eczema were each accepted as equivalent to the dataset label “eczema-spongiotic-dermatitis”; other inflammatory dermatoses required exact entity-level matching. Bowen disease was accepted as equivalent to “squamous-cell-carcinoma-in-situ”. Keratoacanthoma, including predictions worded as keratoacanthoma/squamous cell carcinoma, was scored within “scc_family.” Spitz nevus was scored within “benign_melanocytic_family.” Inflammatory breast cancer was accepted as equivalent to “metastatic-carcinoma,” given the clinically and visually indistinguishable dermal presentation (carcinoma erysipeloides pattern) and the inability to differentiate dermal lymphatic carcinoma invasion from cutaneous carcinoma metastases on clinical imaging alone. Benign longitudinal melanonychia and ethnic melanonychia were accepted as equivalent to “hyperpigmentation,” reflecting melanocytic activation with increased melanin deposition without melanocytic proliferation.

Angioma equivalences. For the ground-truth entry “angioma,” the following predictions were accepted: angioma, cherry angioma, cherry hemangioma, hemangioma, and capillary hemangioma. Angiokeratoma, glomangioma/glomus tumor, pyogenic granuloma/lobular capillary hemangioma, and arteriovenous hemangioma were retained as separate entities and were not credited unless exactly matched.

Benign melanocytic family scope. Accepted predictions within “benign_melanocytic_family” included blue nevus/nevi, dysplastic or atypical nevi, Spitz/Reed nevi, and intradermal, compound, junctional, acral melanocytic, and speckled lentiginous nevi. Non-melanocytic entities containing the word “nevus” were not credited unless explicitly mapped within this family.

Spelling variants. Minor spelling variants were accepted when unambiguous and not diagnosis-altering. For example, “intrademal nevus” was scored as equivalent to “intradermal nevus.”
