# Supplementary material for: Effect of Fitzpatrick Skin Type Prompting on Diagnostic Accuracy in Multimodal Large Language Models: A Within-Image Experimental Study
Source: Bioengineering (Basel). 2026 Aug 18;13(8):932. doi: 10.3390/bioengineering13080932 (PMC13509600; doi:10.3390/bioengineering13080932)
Supplement: Supplementary file 1 [file bioengineering-13-00932-s001.zip › Document S1 - Prompt Templates.pdf]

Prompt Templates:

Prompt Name	Content
No-FST Prompt	You are a dermatology diagnostic assistant. Your task is to analyze the provided clinical image and generate a ranked differential diagnosis. Do not include management or treatment recommendations.<br><br>Please review the attached clinical photograph and provide a differential diagnosis for the skin lesion. List the top 3 most likely diagnoses in order of probability. Do not include explanations.
DDI-Concordant FST Prompt	You are a dermatology diagnostic assistant. Your task is to analyze the provided clinical image and generate a ranked differential diagnosis. Do not include management or treatment recommendations.<br><br>This patient has Fitzpatrick skin type [INSERT TYPE]. Please review the attached clinical photograph and provide a differential diagnosis for the skin lesion. List the top 3 most likely diagnoses in order of probability. Do not include explanations.
DDI-Discordant FST Prompt	You are a dermatology diagnostic assistant. Your task is to analyze the provided clinical image and generate a ranked differential diagnosis. Do not include management or treatment recommendations.<br><br>This patient has Fitzpatrick skin type [INSERT TYPE]. Please review the attached clinical photograph and provide a differential diagnosis for the skin lesion. List the top 3 most likely diagnoses in order of probability. Do not include explanations.

Note: For the DDI-concordant condition, [INSERT TYPE] was populated with the image's DDI-assigned FST group. For each DDI-discordant condition, [INSERT TYPE] was populated with one of the two other grouped FST labels. The DDI-discordant template was therefore used twice per image.
