# Supplementary material for: Effect of Fitzpatrick Skin Type Prompting on Diagnostic Accuracy in Multimodal Large Language Models: A Within-Image Experimental Study
Source: Bioengineering (Basel). 2026 Aug 18;13(8):932. doi: 10.3390/bioengineering13080932 (PMC13509600; doi:10.3390/bioengineering13080932)
Supplement: Supplementary file 1 [file bioengineering-13-00932-s001.zip › Document S2 - Predefined Scoring Ontology.pdf]

Scoring Rules/Criteria:

- Each Ground_Truth_Diagnosis (GT) is mapped to a predefined canonical category according to the study ontology
- Only predefined subtype families (melanoma, SCC, BCC, benign melanocytic, seborrheic keratosis, verruca), plus the explicitly listed entity-level equivalences below, were collapsed for scoring
- Diagnoses within the same predefined collapsed subtype family are considered equivalent for correctness
- All other diagnoses require exact entity-level matching
- Only the top three ranked predictions are evaluated
- The first ranked prediction that matches the GT canonical category determines the score; evaluation stops at the first match
- Rank-based scoring is assigned as: Rank 1 = 3 points; Rank 2 = 2 points; Rank 3 = 1 point; not present in top 3 = 0 points
- A prediction is considered correct if it maps to the same canonical category as the GT
- A prediction is considered incorrect if it maps to a different canonical entity
- Diagnoses are not considered correct solely due to belonging to the same broad clinical class (e.g., infection, inflammatory, vascular)
- Collapsing across malignant and benign entities is not permitted
- Subtype specificity does not override rank position

Predefined Ontology:

**Ground_Truth_Diagnosis**	**Canonical_Category**	**Collapsing_Type**
melanoma-in-situ	melanoma_family	Collapsed_Subtype_Family
mycosis-fungoides	mycosis-fungoides	Exact_Entity_Level
squamous-cell-carcinoma-in-situ	scc_family	Collapsed_Subtype_Family
basal-cell-carcinoma	bcc_family	Collapsed_Subtype_Family
squamous-cell-carcinoma	scc_family	Collapsed_Subtype_Family
melanoma-acral-lentiginous	melanoma_family	Collapsed_Subtype_Family
basal-cell-carcinoma-superficial	bcc_family	Collapsed_Subtype_Family
squamous-cell-carcinoma-keratoacanthoma	scc_family	Collapsed_Subtype_Family
subcutaneous-t-cell-lymphoma	subcutaneous-t-cell-lymphoma	Exact_Entity_Level
melanocytic-nevi	benign_melanocytic_family	Collapsed_Subtype_

		Family
seborrheic-keratosis-irritated	seborrheic_keratosis_family	Collapsed_Subtype_Family
focal-acral-hyperkeratosis	focal-acral-hyperkeratosis	Exact_Entity_Level
hyperpigmentation	hyperpigmentation	Exact_Entity_Level
lipoma	lipoma	Exact_Entity_Level
foreign-body-granuloma	foreign-body-granuloma	Exact_Entity_Level
blue-nevus	benign_melanocytic_family	Collapsed_Subtype_Family
verruca-vulgaris	verruca_family	Collapsed_Subtype_Family
acrochordon	acrochordon	Exact_Entity_Level
wart	verruca_family	Collapsed_Subtype_Family
abrasions-ulcerations-and-physical-injuries	abrasions-ulcerations-and-physical-injuries	Exact_Entity_Level
basal-cell-carcinoma-nodular	bcc_family	Collapsed_Subtype_Family
epidermal-cyst	epidermal-cyst	Exact_Entity_Level
acquired-digital-fibrokeratoma	acquired-digital-fibrokeratoma	Exact_Entity_Level
epidermal-nevus	epidermal-nevus	Exact_Entity_Level
seborrheic-keratosis	seborrheic_keratosis_family	Collapsed_Subtype_Family
trichilemmoma	trichilemmoma	Exact_Entity_Level
pyogenic-granuloma	pyogenic-granuloma	Exact_Entity_Level
neurofibroma	neurofibroma	Exact_Entity_Level
syringocystadenoma-papilliferum	syringocystadenoma-papilliferum	Exact_Entity_Level
nevus-lipomatosus-superficialis	nevus-lipomatosus-superficialis	Exact_Entity_Level
benign-keratosis	seborrheic_keratosis_family	Collapsed_Subtype_Family
inverted-follicular-keratosis	seborrheic_keratosis_family	Collapsed_Subtype_Family
onychomycosis	onychomycosis	Exact_Entity_Level
dermatofibroma	dermatofibroma	Exact_Entity_Level
trichofolliculoma	trichofolliculoma	Exact_Entity_Level
lymphocytic-infiltrations	lymphocytic-infiltrations	Exact_Entity_Level
prurigo-nodularis	prurigo-nodularis	Exact_Entity_Level
kaposi-sarcoma	kaposi-sarcoma	Exact_Entity_Level
scar	scar	Exact_Entity_Level

eccrine-poroma	eccrine-poroma	Exact_Entity_Level
angioleiomyoma	angioleiomyoma	Exact_Entity_Level
keloid	keloid	Exact_Entity_Level
hematoma	hematoma	Exact_Entity_Level
metastatic-carcinoma	metastatic-carcinoma	Exact_Entity_Level
melanoma	melanoma_family	Collapsed_Subtype_Family
angioma	angioma	Exact_Entity_Level
folliculitis	folliculitis	Exact_Entity_Level
atypical-spindle-cell-nevus-of-reed	benign_melanocytic_family	Collapsed_Subtype_Family
xanthogranuloma	xanthogranuloma	Exact_Entity_Level
eczema-spongiotic-dermatitis	eczema-spongiotic-dermatitis	Exact_Entity_Level
arteriovenous-hemangioma	arteriovenous-hemangioma	Exact_Entity_Level
acne-cystic	acne-cystic	Exact_Entity_Level
verruciform-xanthoma	verruciform-xanthoma	Exact_Entity_Level
molluscum-contagiosum	molluscum-contagiosum	Exact_Entity_Level
condyloma-accuminatum	condyloma-accuminatum	Exact_Entity_Level
morphea	morphea	Exact_Entity_Level
neuroma	neuroma	Exact_Entity_Level
dysplastic-nevus	benign_melanocytic_family	Collapsed_Subtype_Family
nodular-melanoma-(nm)	melanoma_family	Collapsed_Subtype_Family
actinic-keratosis	actinic-keratosis	Exact_Entity_Level
pigmented-spindle-cell-nevus-of-reed	benign_melanocytic_family	Collapsed_Subtype_Family
dermatomyositis	dermatomyositis	Exact_Entity_Level
glomangioma	glomangioma	Exact_Entity_Level
cellular-neurothekeoma	cellular-neurothekeoma	Exact_Entity_Level
fibrous-papule	fibrous-papule	Exact_Entity_Level
graft-vs-host-disease	graft-vs-host-disease	Exact_Entity_Level
lichenoid-keratosis	seborrheic_keratosis_family	Collapsed_Subtype_Family
reactive-lymphoid-hyperplasia	reactive-lymphoid-hyperplasia	Exact_Entity_Level
coccidioidomycosis	coccidioidomycosis	Exact_Entity_Level
leukemia-cutis	leukemia-cutis	Exact_Entity_Level
sebaceous-carcinoma	sebaceous-carcinoma	Exact_Entity_Level
chondroid-syringoma	chondroid-syringoma	Exact_Entity_Level

tinea-pedis	tinea-pedis	Exact_Entity_Level
solar-lentigo	solar-lentigo	Exact_Entity_Level
clear-cell-acanthoma	clear-cell-acanthoma	Exact_Entity_Level
abscess	abscess	Exact_Entity_Level
blastic-plasmacytoid-dendritic-cell-neoplasm	blastic-plasmacytoid-dendritic-cell-neoplasm	Exact_Entity_Level
acral-melanotic-macule	benign_melanocytic_family	Collapsed_Subtype_Family

Additional Notes:

- Verruca plana, verruca plantaris, and verruca filiformis were considered in verruca_family
- Inflammatory breast cancer was considered equivalent to metastatic-carcinoma based on their clinically and visually indistinguishable dermal presentation (carcinoma erysipeloides pattern), acknowledging that IBC represents dermal lymphatic invasion by carcinoma and cannot be differentiated from cutaneous carcinoma metastases on clinical imaging alone.
- Benign longitudinal melanonychia and ethnic melanonychia were considered equivalent to hyperpigmentation because they represent melanocytic activation resulting in increased melanin deposition without melanocytic proliferation
- Spitz nevus was considered benign_melanocytic_family
- Seborrheic keratosis was considered equivalent to inverted-follicular-keratosis
- Seborrheic dermatitis, nummular dermatitis, and nummular eczema were considered equivalent to eczema-spongiotic-dermatitis for scoring; other inflammatory dermatoses were not collapsed unless they exactly matched the ground-truth entity
- Dermatosis papulosa nigra was considered within the seborrheic_keratosis_family for scoring
- Keratoacanthoma, including predictions worded as keratoacanthoma/squamous cell carcinoma, was considered within the scc_family for scoring
- Bowen disease was considered equivalent to squamous-cell-carcinoma-in-situ
- Minor spelling variants were accepted when unambiguous and not diagnosis-altering; for example, “intrademal nevus” was scored as “intradermal nevus”
- Benign melanocytic-family predictions included blue nevus/nevi, dysplastic or atypical nevi, Spitz/Reed nevi, intradermal, compound, junctional, acral melanocytic, and speckled lentiginous nevi; non-melanocytic entities containing “nevus” were not credited unless explicitly mapped.
- For ground-truth angioma, angioma, cherry angioma, cherry hemangioma, hemangioma, and capillary hemangioma were accepted; angiokeratoma, glomangioma/glomus tumor, pyogenic granuloma/lobular capillary hemangioma, and arteriovenous hemangioma were retained as separate entities unless exactly matched.
