# Supplementary material for: Effect of Fitzpatrick Skin Type Prompting on Diagnostic Accuracy in Multimodal Large Language Models: A Within-Image Experimental Study
Source: Bioengineering (Basel). 2026 Aug 18;13(8):932. doi: 10.3390/bioengineering13080932 (PMC13509600; doi:10.3390/bioengineering13080932)
Supplement: Supplementary file 1 [file bioengineering-13-00932-s001.zip › Table S1 - Post Hoc Equal-Image-Weighted Sensitivity CLMM.pdf]

Table S1. Primary and Post Hoc Equal-Image-Weighted Sensitivity CLMM Results

Comparison	Primary CLMM OR (95% CI)	Primary FDR-adjusted P	Weighted Sensitivity CLMM OR (95% CI)	Sensitivity Nominal P
Gemini 3.1 Pro configuration vs. ChatGPT 5.2 configuration	2.32 (2.01–2.67)	<.001	2.25 (1.94–2.61)	<.001
DDI-concordant FST vs. no-FST	1.16 (0.95–1.40)	.283	1.15 (0.95–1.40)	.145
Adjacent DDI-discordant FST vs. no-FST	0.99 (0.82–1.18)	.893	0.98 (0.81–1.19)	.857
Extreme DDI-discordant FST vs. no-FST	0.88 (0.70–1.12)	.398	0.93 (0.73–1.17)	.526

Note: The primary CLMM was prespecified and included model configuration and prompt condition as fixed effects with image as a random intercept. The weighted sensitivity CLMM was post hoc, assigned weight 0.5 to each of the two adjacent DDI-discordant observations from FST III–IV images and weight 1 to all other observations, and additionally adjusted for DDI-assigned FST group. All 5,248 records were retained; total case weight was 4,766. Sensitivity P values are nominal. CI, confidence interval; CLMM, cumulative link mixed model; DDI, Diverse Dermatology Images; FDR, false discovery rate; FST, Fitzpatrick skin type; OR, odds ratio.
