# Supplementary material for: Effect of Fitzpatrick Skin Type Prompting on Diagnostic Accuracy in Multimodal Large Language Models: A Within-Image Experimental Study
Source: Bioengineering (Basel). 2026 Aug 18;13(8):932. doi: 10.3390/bioengineering13080932 (PMC13509600; doi:10.3390/bioengineering13080932)
Supplement: Supplementary file 1 [file bioengineering-13-00932-s001.zip › Table S2 - Post Hoc Descriptive Top-1 and Top-3 Accuracy.pdf]

Table S2. Post Hoc Descriptive Top-1 and Top-3 Diagnostic Accuracy

Model Configuration	Prompt Condition	Eligible Images, n	Top-1 Accuracy (%)	Top-3 Diagnostic Inclusion Accuracy (%)
ChatGPT 5.2	No-FST	656	23.0	45.4
ChatGPT 5.2	DDI-concordant FST	656	25.3	46.8
ChatGPT 5.2	Adjacent DDI-discordant FST	656	22.7	46.0
ChatGPT 5.2	Extreme DDI-discordant FST	415	17.3	38.8
Gemini 3.1 Pro	No-FST	656	31.6	53.5
Gemini 3.1 Pro	DDI-concordant FST	656	32.6	54.4
Gemini 3.1 Pro	Adjacent DDI-discordant FST	656	31.9	52.6
Gemini 3.1 Pro	Extreme DDI-discordant FST	415	25.1	45.5

Note: Top-1 accuracy was defined as the biopsy-confirmed diagnosis or accepted ontology equivalent ranked first. Top-3 diagnostic inclusion accuracy was defined as its presence anywhere in the three-item differential. For DDI FST III–IV images, the two adjacent DDI-discordant binary outcomes were averaged within image so that each image contributed equal total weight. Extreme DDI-discordant rows were restricted to the 415 outer-group images. No additional hypothesis testing was performed.
