# Supplementary material for: Effect of Fitzpatrick Skin Type Prompting on Diagnostic Accuracy in Multimodal Large Language Models: A Within-Image Experimental Study
Source: Bioengineering (Basel). 2026 Aug 18;13(8):932. doi: 10.3390/bioengineering13080932 (PMC13509600; doi:10.3390/bioengineering13080932)
Supplement: Supplementary file 1 [file bioengineering-13-00932-s001.zip › Table S3 - Post Hoc Binary Malignancy Detection.pdf]

Table S3. Post Hoc Binary Malignancy Detection by Model Configuration and Original Prompt Submission

Model	Prompt condition	TP	FP	TN	FN	Sensitivity, % (95% CI)	Specificity, % (95% CI)	PPV, % (95% CI)	NPV, % (95% CI)	F1, %	Accuracy, % (95% CI)
ChatGPT 5.2	No-FST	115	279	206	56	67.3 (59.9–73.8)	42.5 (38.2–46.9)	29.2 (24.9–33.9)	78.6 (73.3–83.2)	40.7	48.9 (45.1–52.8)
ChatGPT 5.2	DDI-concordant FST	113	277	208	58	66.1 (58.7–72.8)	42.9 (38.6–47.3)	29.0 (24.7–33.7)	78.2 (72.9–82.7)	40.3	48.9 (45.1–52.8)
ChatGPT 5.2	DDI-discordant FST prompt 1	125	329	156	46	73.1 (66.0–79.2)	32.2 (28.2–36.4)	27.5 (23.6–31.8)	77.2 (71.0–82.5)	40.0	42.8 (39.1–46.7)
ChatGPT 5.2	DDI-discordant FST prompt 2	100	246	239	71	58.5 (51.0–65.6)	49.3 (44.9–53.7)	28.9 (24.4–33.9)	77.1 (72.1–81.4)	38.7	51.7 (47.9–55.5)
Gemini 3.1 Pro	No-FST	139	374	111	32	81.3 (74.8–86.4)	22.9 (19.4–26.8)	27.1 (23.4–31.1)	77.6 (70.1–83.7)	40.6	38.1 (34.5–41.9)
Gemini 3.1 Pro	DDI-concordant FST	135	381	104	36	78.9 (72.2–84.4)	21.4 (18.0–25.3)	26.2 (22.6–30.1)	74.3 (66.5–80.8)	39.3	36.4 (32.8–40.2)
Gemini 3.1 Pro	DDI-discordant FST prompt 1	143	393	92	28	83.6 (77.4–88.4)	19.0 (15.7–22.7)	26.7 (23.1–30.6)	76.7 (68.3–83.3)	40.5	35.8 (32.2–39.6)
Gemini 3.1 Pro	DDI-discordant FST prompt 2	132	370	115	39	77.2 (70.3–82.8)	23.7 (20.1–27.7)	26.3 (22.6–30.3)	74.7 (67.3–80.9)	39.2	37.7 (34.0–41.4)

Note: A response was malignancy-positive when at least one top-three diagnosis matched an explicit malignant entity or family and negative otherwise. Premalignant or benign terms, including actinic keratosis, dysplastic nevus, Bowenoid papulosis, and Spitz/Reed nevus, were negative unless an explicit malignancy was also listed. The DDI malignancy field was the reference standard. Wilson 95% confidence intervals are shown for proportions; F1 is reported without a confidence interval. These analyses were post hoc and descriptive. CI, confidence interval; DDI, Diverse Dermatology Images; FN, false negative; FP, false positive; NPV, negative predictive value; PPV, positive predictive value; TN, true negative; TP, true positive.
